# Phase 2 Trial of Combination Radiotherapy and Pembrolizumab Plus Chemotherapy in Patients With Previously Untreated Metastatic NSCLC: NJLCG 1902

**DOI:** 10.1016/j.jtocrr.2025.100817

**Published:** 2025-02-28

**Authors:** Yoko Tsukita, Rei Umezawa, Taku Nakagawa, Akira Anbai, Tomonori Makiguchi, Hisashi Tanaka, Yosuke Horii, Aya Suzuki, Ryo Morita, Hitomi Nogawa, Hiroshi Yokouchi, Nozomu Kimura, Keiichi Jingu, Akira Inoue, Hisatoshi Sugiura, Eisaku Miyauchi

**Affiliations:** a Department of Respiratory Medicine, Tohoku University Graduate School of Medicine, Sendai, Japan; b Department of Radiation Oncology, Tohoku University Graduate School of Medicine, Sendai, Japan; c Department of Thoracic Surgery, Omagari Kosei Medical Center, Daisen, Japan; d Department of Radiology, Omagari Kosei Medical Center, Daisen, Japan; e Department of Respiratory Medicine, Hirosaki University Graduate School of Medicine, Hirosaki, Japan; f Division of Pulmonary Medicine, Department of Internal Medicine, Iwate Medical University School of Medicine, Morioka, Japan; g Department of Respiratory Medicine, Miyagi Cancer Center, Natori, Japan; h Department of Respiratory Medicine, Akita Kousei Medical Center, Akita, Japan; i Department of Respiratory Medicine, Yamagata Prefectural Central Hospital, Yamagata, Japan; j Department of Respiratory Medicine, NHO Hokkaido Cancer Center, Sapporo, Japan; k Department of Palliative Medicine, Tohoku University School of Medicine, Sendai, Japan

**Keywords:** NSCLC, Radiotherapy, Abscopal, Pembrolizumab, Chemotherapy

## Abstract

**Introduction:**

Treatment strategies that enhance the efficacy of immunotherapy are desired. Radiotherapy can enhance immunity, but the utility of adding radiotherapy to immunotherapy plus platinum-doubled chemotherapy in patients with metastatic NSCLC has not been explored.

**Methods:**

This multicenter, single-arm phase 2 trial evaluated the efficacy and safety of combining radiotherapy with pembrolizumab plus chemotherapy in patients with previously untreated metastatic NSCLC. Patients begin receiving pembrolizumab plus platinum-doublet chemotherapy within 1 week of starting radiotherapy (30 Gy in 10 fractions). The primary end point was the 12-month progression-free survival (PFS) rate. The secondary end points included PFS, overall survival, and toxicity profiles.

**Results:**

Forty patients were enrolled. In total, 37 and 38 patients were analyzed for efficacy and safety, respectively. The 12-month PFS rate was 44.3% (90% confidence interval [CI]: 30.3–57.3), which met the primary end point. The median PFS was 8.4 months (95% CI: 5.7–22.2), and the median overall survival was 30.1 months (95% CI: 22.3–not reached). Grade 3 or 4 adverse events occurred in 25 patients (65.8%), and one treatment-related death was reported. Pneumonitis was reported in 10 patients (26.3%), including two cases of grade 3 pneumonitis and one case of grade 5.

**Conclusions:**

Adding radiotherapy to pembrolizumab plus platinum-doublet chemotherapy led to promising efficacy in patients with previously untreated metastatic NSCLC. Although caution should be exercised with regard to pneumonitis, adverse events were tolerable. Further research is needed to confirm the efficacy and safety of this strategy.

## Introduction

Immune checkpoint inhibitors (ICIs) represent a breakthrough in the treatment of NSCLC without targetable oncogene alterations. Treatment with pembrolizumab, a monoclonal antibody targeting programmed death-1, resulted in improved overall survival (OS) compared with chemotherapy for programmed death-ligand 1 (PD-L1) positive NSCLC in the first and second-line settings.^1^^,^^2^ Phase 3 studies found that the addition of pembrolizumab to platinum-doublet chemotherapy resulted in longer OS and progression-free survival (PFS) relative to chemotherapy alone, regardless of PD-L1 tumor proportion score (TPS).^3^^,^^4^ Nevertheless, many patients do not achieve long-term survival owing to primary or acquired resistance to ICIs.^5^ Treatment strategies to enhance the effectiveness of ICIs are needed.

Radiotherapy increases the release of tumor-associated antigens and cross-presentation by antigen-presenting cells and consequently promotes dendritic cell function, T-cell priming, and infiltration into tumor deposits. This activation of the immune system can result in an antitumor effect outside of the radiation field, known as the abscopal effect.^6^^,^^7^ A combination of radiotherapy and immunotherapy provides an opportunity to boost the abscopal effect.^8^ Several studies have evaluated the efficacy and safety of adding radiotherapy to immunotherapy for metastatic NSCLC. In a secondary analysis of the KEYNOTE-001 data set, previous radiotherapy treatment in patients with advanced NSCLC resulted in longer PFS and OS with pembrolizumab treatment than in patients who did not receive previous radiotherapy, with an acceptable safety profile.^9^ Two randomized trials comparing pembrolizumab versus pembrolizumab with radiotherapy in advanced NSCLC reported a clinically meaningful higher response rate and prolonged PFS, although this was not statistically significant.^10^^,^^11^ Another randomized trial comparing durvalumab plus tremelimumab alone or in combination with low-dose or hypofractionated radiotherapy in metastatic NSCLC refractory to previous PD-L1 therapy was terminated owing to futility in an interim analysis.^12^ In locally advanced stage Ⅲ NSCLC, concurrent pembrolizumab plus chemoradiation therapy reported a promising antitumor activity and manageable safety profile in the phase 2, two-cohort nonrandomized KEYNOTE-799 study.^13^

Nevertheless, the efficacy and safety of adding radiation to immunotherapy plus platinum-doublet chemotherapy, which is one of the current metastatic NSCLC standard regimens, has not been explored. Cytotoxic agents may also affect the tumor microenvironment by activating immune cells through the release of damage-related molecular patterns.^14^

Therefore, we conducted a multicenter phase 2 trial to evaluate the efficacy and safety of combining radiotherapy with pembrolizumab plus platinum-doublet chemotherapy for previously untreated patients with advanced NSCLC.

## Materials and Methods

### Patient Selection

This open-label, multicenter, single-arm, phase 2 trial was conducted in accordance with the Helsinki Declaration of the World Medical Association. The study protocol was approved by the institutional review boards of Tohoku University Hospital and each institution. Eligible patients had the following characteristics: cytologically- or histologically-confirmed NSCLC; clinical stage IIIB, IIIC without indications for definitive thoracic radiotherapy, stage IV, or postoperative recurrent disease; scheduled to receive pembrolizumab in combination with a cytotoxic agent as first-line therapy; and lesions that could be irradiated at a total dose of 30 Gy in 10 fractions. Further eligibility criteria were the following: aged 20 years or above, an Eastern Cooperative Oncology Group performance status of 0 to 1, measurable disease except radiation site according to the Response Evaluation Criteria in Solid Tumors version 1.1, adequate organ function (neutrophil count ≥1500/mm^3^, platelets ≥100,000/mm^3^, hemoglobin ≥9.0 g/dL, serum bilirubin ≤1.5-fold the upper limit of normal, aspartate aminotransferase and alanine aminotransferase ≤2.5-fold the upper limit of normal, creatinine clearance level ≥45 mL/min, and arterial oxygen pressure ≥60 mmHg or percutaneous oxygen saturation ≥95%), an estimated life expectancy of three months or longer. The exclusion criteria included *EGFR*, *ALK*, *ROS1* or *BRAF*-mutant, previous treatment with radiotherapy or immunotherapy, symptomatic brain metastasis, history of autoimmune disease or organ transplantation, use of systemic steroid treatment or immunosuppressive agents, interstitial lung disease, massive effusion requiring drainage, active infection, severe comorbidities such as cardiac disease or uncontrolled diabetes, history of synchronous or metachronous malignancies, and pregnancy. Written informed consent was obtained from all patients.

### Procedures and Outcomes

Patients were scheduled to receive radiotherapy at a total dose of 30 Gy in 10 fractions 5 days per week. Three-dimensional conformal radiotherapy was delivered using a 4 megavolt or higher photon beam from a medical linear accelerator. Treatment planning by computed tomography (CT) was performed, with the slice thickness of the CT scan being 5 mm or smaller. The gross target volume (GTV) was defined as a macroscopic lesion identifiable by CT. Not including all target lesion of GTV was allowed if dose constraints were not met. The clinical target volume was defined as identical to the GTV. The planning target volume (PTV) was defined as the clinical target volume plus at least a 5-mm margin. Considering the patients’ clinical symptoms and the risk of radiation pneumonitis, the priority lesions for irradiation were as follows: (1) lesions that need to be irradiated clinically (symptomatic or the possibility of future symptoms), (2) lesions with the largest tumor volume outside the lung, and (3) lesions with the largest tumor volume within the lung; a maximum of two lesions were allowed. The lesions to be irradiated were decided after discussions between each patient's respiratory medicine physician and radiation oncologist. The tumor volume was assessed by radiation oncologists at each institution using a radiation treatment planning system. The constraints were as follows: PTV between 50 cm^3^ and 400 cm^3^ and the lung V20 being 20% or less if thoracic irradiation was selected. Because 30 Gy in 10 fractions is usually indicated for palliative treatment, such as irradiating bone metastases in metastatic NSCLC, we considered that the 30 Gy irradiation dose was more feasible and easier to generalize.

Patients began receiving systemic therapy within 1 week of the beginning of radiation therapy. Patients with nonsquamous carcinoma received four cycles of intravenously administered cisplatin (75 mg/m^2^) or carboplatin (area under the curve 5) plus pemetrexed (500 mg/m^2^) plus pembrolizumab (200 mg/body) every 3 weeks, followed by pemetrexed plus pembrolizumab every 3 weeks. Patients with squamous cell carcinoma received four cycles of carboplatin (area under the curve 6) plus pembrolizumab (200 mg/body) on day 1 and either paclitaxel (100 mg/m^2^) on day 1 or nab-paclitaxel on days 1, 8, and 15. All treatments were administered intravenously in 3-week cycles, followed by pembrolizumab every three weeks.

Treatment was continued until disease progression, death, unacceptable toxicity, investigator decision, or treatment refusal. Any requirement for dose reduction was judged by the attending physicians. Pembrolizumab was discontinued if treatment was interrupted for more than 12 weeks, cytotoxic agents were discontinued if treatment was interrupted for more than 6 weeks, and the protocol treatment was discontinued if all agents were discontinued.

Baseline assessment included patient characteristics, physical examination, complete blood count, biochemistry, and thoracoabdominal CT. Tumor measurements were performed by thoracoabdominal CT every 6 to 10 weeks (until month 6) and every nine to 12 weeks thereafter until disease progression. A central review procedure was employed to evaluate the tumor response.

The primary end point was 12-month PFS from the date of registration. Secondary end points were PFS, OS, overall response rate (ORR), disease control rate (DCR), and toxicity profiles. ORR and DCR were defined as the proportion of patients achieving a complete or partial response, and complete response, partial response, or stable disease, respectively, as measured at nonirradiated sites, according to Response Evaluation Criteria in Solid Tumors version 1.1. PFS was defined as the period from the date of registration to disease progression or death from any cause or the last day of follow-up. OS was defined as the period from the date of registration to death from any cause or the last day of follow-up. All response evaluations were validated by independent extramural review. Adverse events (AEs) were graded according to the National Cancer Institute-Common Toxicity Criteria version 5.0.

### Statistical Analysis

We assessed responses in patients who received at least one dose of the study drugs and safety in patients who received at least one dose of the study drugs or one fraction of radiotherapy. In the KEYNOTE-189 and KEYNOTE-407 trials, the 12-month PFS rates were 34.1% (95% confidence interval [CI]: 28.8%–39.5%) and 31.3% (95% CI: 24.1%–38.7%), respectively. As the lower limit of the 95% CI was 28.8% to 24.1%, the threshold had to be greater than 28.8% and was thus set at 30%. Assuming a 12-month PFS rate of 50% in eligible patients as indicative of potential usefulness and 30% as the lower limit of interest, with a one-sided significance level of 0.05 and a power of 80%, the required total sample size was calculated as 37. Considering potential ineligible patients after enrollment, the sample recruitment size was set at 40. The 12-month PFS rates were estimated using the Kaplan-Meier method, and the 90% CIs were calculated. We estimated the median PFS and OS using the Kaplan-Meier method. Cox proportional hazard models were used to determine hazard ratios for PFS and OS. Graphpad Prism, Stata 13, and JMP Pro 17 were used for statistical analyses. This trial was registered as UMIN 000036856.

## Results

### Patient Characteristics

**Figure 1:**
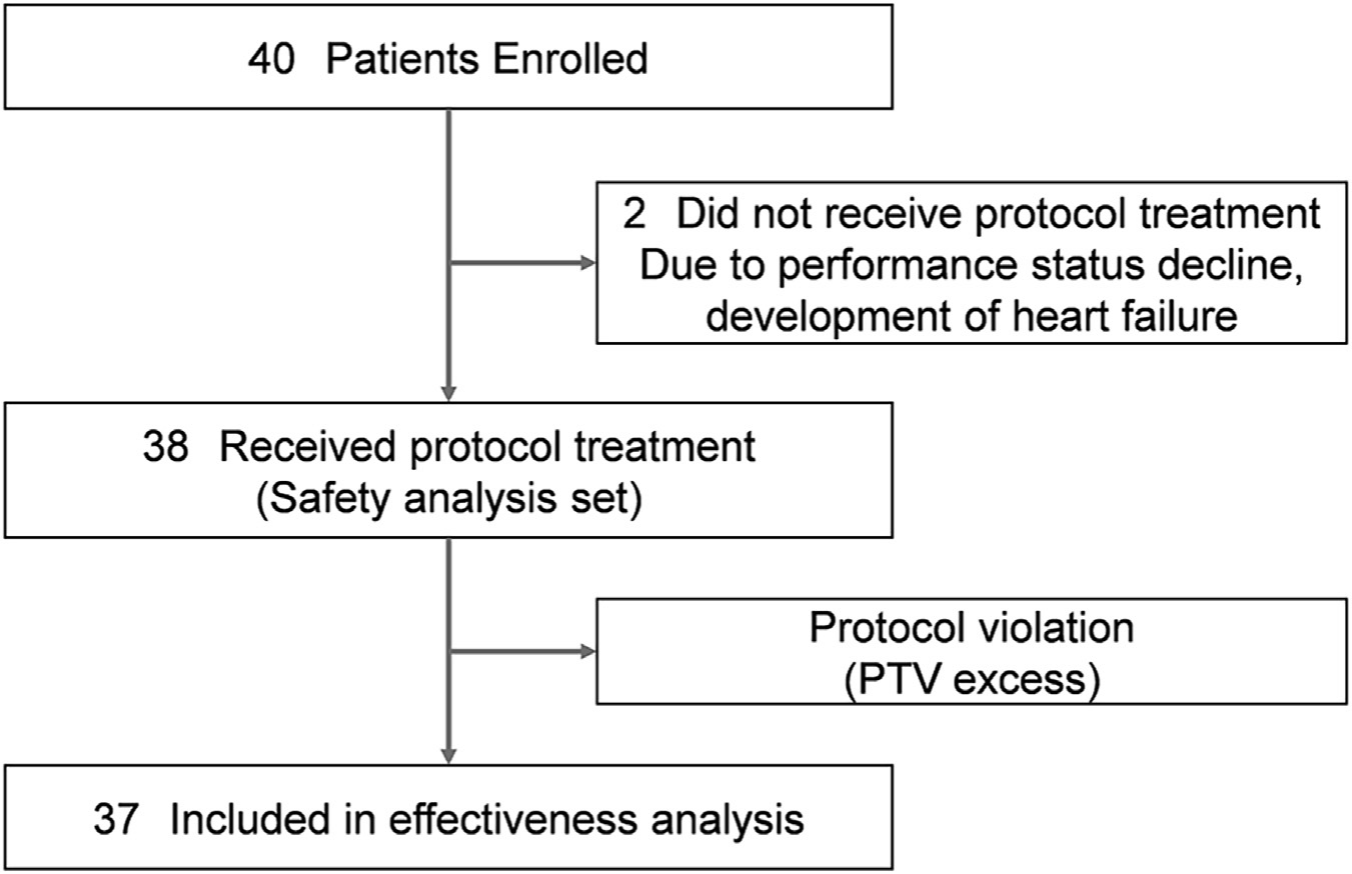
Patient flow diagram. PTV, planning target volume.

A total of 40 patients were enrolled from July 2019 to May 2023 from eight institutions in Japan. Two patients did not receive the protocol treatment owing to performance status decline in one and the development of heart failure in the other. One patient was excluded from the efficacy analysis because they did not meet the eligibility criteria owing to PTV excess. Thus, data from 37 patients could be analyzed for efficacy and 38 for safety (Fig. 1). The characteristics of patients who received at least one dose of study therapy are shown in Table 1. Their median age was 68 years, with 58% and 42% having an Eastern Cooperative Oncology Group performance status of 0 and 1, respectively. Seventy-six percent of the patients had adenocarcinoma, and 21% had squamous cell carcinoma. The PD-L1 TPS was less than 1%, 1% to 49%, 50% or higher, and unknown in 16%, 37%, 37%, and 11% of patients, respectively. The most common sites of radiotherapy delivered on protocol were bones (53%), followed by lymph nodes (21%) and lungs (13%). Seventeen cases were irradiated at symptomatic sites, including 16 at bones and one at mediastinal lymph nodes.

**Table 1:** Baseline Characteristics of the Patients

Characteristics	n (%) (n = 38)
Age, median (range) (y)	68 (47–81)
Sex	
Male	29 (76.3)
Female	9 (23.7)
ECOG performance status	
0	22 (57.9)
1	16 (42.1)
Smoking history	
Never	2 (5.3)
Current or former	36 (94.7)
Histology	
Adenocarcinoma	29 (76.3)
Squamous cell carcinoma	8 (21.1)
NSCLC NOS	1 (2.6)
Disease stage	
IIIC	1 (2.6)
IVA	11 (28.9)
IVB	25 (65.8)
Postoperative recurrence	1 (2.6)
PD-L1 tumor proportion score	
<1%	6 (15.8)
1%–49%	14 (36.8)
≥50%	14 (36.8)
Unknown	4 (10.5)
Regimen	
Carboplatin, pemetrexed, pembrolizumab	19 (50.0)
Cisplatin, pemetrexed, pembrolizumab	11 (28.9)
Carboplatin, nab-paclitaxel, pembrolizumab	7 (18.4)
Carboplatin, paclitaxel, pembrolizumab	1 (2.6)
Radiation site	
Bone	20 (52.6)
Lymph node	8 (21.1)
Lung	5 (13.2)
Adrenal grand	3 (7.9)
Other	2 (5.3)

ECOG, Eastern Cooperative Oncology Group; NSCLC NOS, NSCLC not otherwise specified; PD-L1, programmed death-ligand 1.

### Efficacy

**Figure 2:**
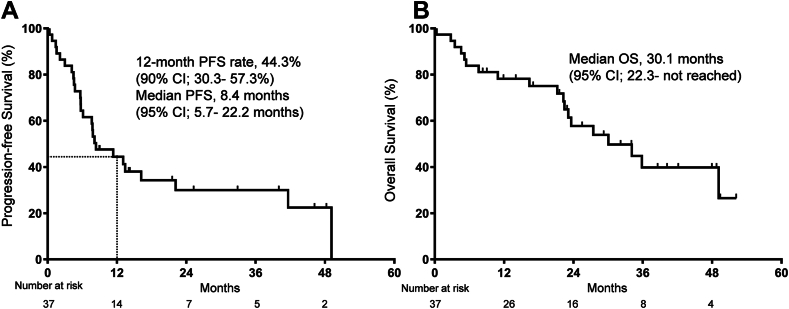
*(A)* Kaplan-Meier curves for PFS. *(B)* Kaplan-Meier curves for OS. CI, confidence interval; OS, overall survival; PFS, progression-free survival.

At the time of data cutoff (January 2024), three patients were continuing treatment, and 20 had died. The median follow-up duration was 22.9 months (range: 0.30–52.2 mo). All patients completed the radiotherapy on protocol. The median time from the beginning of radiation therapy to drug therapy was 1 day (range: 0-6 d). Twenty-seven patients experienced a PFS event, and 14 remained progression-free at 12 months. The primary end point of 12-month PFS was achieved in 44.3% (90% CI: 30.3%–57.3%) (Fig. 2A). The median PFS was estimated to be 8.4 months (95% CI: 5.7–22.2), and the median OS was 30.1 months (95% CI: 22.3–not reached) (Fig. 2A and B). The ORR was 67.6% (95% CI: 50.2–82.0), and the DCR was 89.2% (95% CI: 74.6–97.0) (Supplementary Table 1). The median numbers of treatment cycles that the patients underwent are shown in Supplementary Table 2.

**Figure 3:**
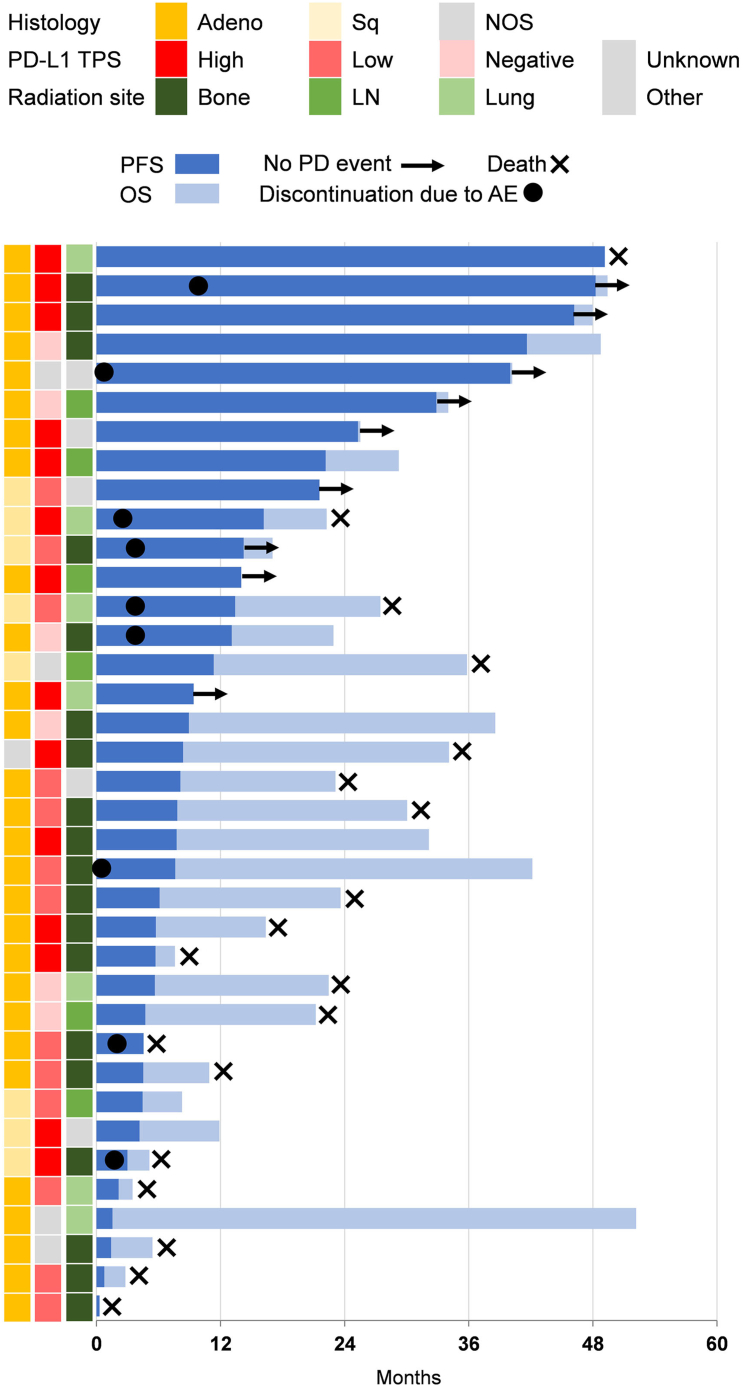
Swimmer plot for individual patients in the effectiveness analysis. Adeno, adeno carcinoma; AE, adverse event; LN, lymph node; NOS, not otherwise specified; OS, overall survival; PD, progression disease; PD-L1 TPS, programmed death-ligand 1 tumor proportion score; PFS, progression-free survival; Sq, squamous cell carcinoma.

The swimmer plot of the 37 patients included in the effectiveness analysis is shown in Figure 3. In the subgroup analyses for PFS and OS, no significant differences were detected, partly owing to the limited sample size (Supplementary Tables 3 and 4).

### Safety

Toxicities are summarized in Table 2. Grade 3 or 4 AEs occurred in 25 patients (65.8%), and grade 5 AEs in two patients (5.3%). One patient developed grade 5 pneumonitis with a potential immunologic cause. The other patient developed grade 5 sudden cardiac arrest. That patient had a history of aortic regurgitation and angina pectoris. The immediate blood test and autopsy imaging did not reveal abnormalities, and the cause of sudden cardiac arrest was thought to be fatal arrhythmia unrelated to treatment. The most common grade 3 or worse AEs were neutropenia (n = 10 [26.3%]), leukopenia (8 [21.1%]), and anemia (6 [15.8%]). Pneumonitis was reported in 10 patients (26.3%), with two patients having grade 3 and one having grade 5. Those with grade 3 or grade 5 pneumonitis had received radiotherapy to the bone but not the lung (Supplementary Table 5). The irradiation field did not include the lungs, and therefore, the attending physicians judged the disease as immune-mediated pneumonitis, not radiation-related pneumonitis. Five of the patients who developed grade 1 or 2 pneumonitis had received radiation to the lung or intrathoracic lymph nodes, with the lung V20 ranging from 1.6% to 11.2% (Supplementary Table 5).

**Table 2:** Adverse Events of Any Cause in 10% or More of Patients Who Received At Least One Dose of Study Therapy

Adverse Event	Grade, n (%)			
	Any	3	4	5
Leukopenia	19 (50.0)	5 (13.2)	3 (7.9)	
Neutropenia	19 (50.0)	6 (15.8)	4 (10.5)	
Anemia	19 (50.0)	6 (15.8)	0 (0)	
Anorexia	19 (50.0)	5 (13.2)	0 (0)	
Thrombocytopenia	14 (36.8)	1 (2.6)	4 (10.5)	
Aspartate or alanine aminotransferase increased	13 (34.2)	3 (7.9)	2 (5.3)	
Rash	11 (28.9)	1 (2.6)	0 (0)	
Pneumonitis	10 (26.3)	2 (5.3)	0 (0)	1 (2.6)
Nausea	8 (21.1)	1 (2.6)	0 (0)	
Hyponatremia	7 (18.4)	0 (0)	1 (2.6)	
Creatinine increased	6 (15.8)	0 (0)	0 (0)	
Constipation	6 (15.8)	0 (0)	0 (0)	
Febrile neutropenia	5 (13.2)	3 (7.9)	2 (5.3)	
Diarrhea	5 (13.2)	0 (0)	0 (0)	
Lung infection	4 (10.5)	4 (10.5)	0 (0)	
Hyperkalemia	4 (10.5)	0 (0)	1 (2.6)	

AEs with potential immunologic causes, as judged by the attending physicians, occurred in 17 patients (44.7%) (Table 3). Of these, 10 (26.3%) had grade 3 or higher AEs. Radiation-mediated AEs, judged by the attending physicians, occurred in six patients (15.8%), three of whom had grade 1 or 2 pneumonitis, and three had esophagitis (one grade 3, two grade 1 or 2). Patients recovered from radiation-mediated AEs with appropriate treatment.

**Table 3:** Adverse Events With an Immune-Related Cause

Adverse Event	Grade, n (%)			
	Any	3	4	5
Pneumonitis	7 (18.4)	2 (5.3)	0 (0)	1 (2.6)
Rash	5 (13.2)	1 (2.6)	0 (0)	0 (0)
Aspartate or alanine aminotransferase increased	4 (10.5)	2 (5.3)	2 (5.3)	0 (0)
Hypothyroidism	2 (5.3)	0 (0)	0 (0)	0 (0)
Creatinine increased	2 (5.3)	0 (0)	0 (0)	0 (0)
Alkaline phosphatase increased	1 (2.6)	1 (2.6)	0 (0)	0 (0)
Colitis	1 (2.6)	1 (2.6)	0 (0)	0 (0)
Allergic reaction	1 (2.6)	1 (2.6)	0 (0)	0 (0)
Hypophysitis	1 (2.6)	1 (2.6)	0 (0)	0 (0)
Myalgia	1 (2.6)	0 (0)	0 (0)	0 (0)

Treatment was discontinued in 11 patients (28.9%) because of AEs owing to pneumonitis (one grade 2, two grade 3 and one grade 5), elevated liver enzymes (two grade 3 and one grade 4), lung infection (one grade 3), hypophysitis (one grade 3), and small intestinal perforation because of metastasis (one grade 4).

## Discussion

We investigated the efficacy and safety of adding radiotherapy to a combination of immunotherapy plus platinum-doublet chemotherapy in patients with previously untreated metastatic NSCLC. At the primary end point, the 12-month PFS rate was met.

Several trials have been conducted to evaluate the addition of radiotherapy to immunotherapy.^10^, ^11^, ^12^^,^^15^, ^16^, ^17^, ^18^, ^19^, ^20^ Two of these were randomized trials,^10^^,^^11^ and in their pooled analysis,^21^ adding radiotherapy to pembrolizumab significantly increased responses and prolonged PFS and OS relative to pembrolizumab alone. In these studies, patients received pembrolizumab monotherapy, and patients with more than two lines of previous chemotherapy were also included. In contrast, our cohort comprised patients scheduled to receive pembrolizumab plus chemotherapy as first-line systemic therapy. The question of the survival benefit of the addition of radiotherapy to the combination of immunotherapy plus platinum-doublet chemotherapy had not been fully explored. To the best of our knowledge, this is the first prospective trial to investigate the efficacy and safety of adding radiotherapy to immunotherapy plus chemotherapy in previously untreated metastatic NSCLC patients. In the KEYNOTE-189 trial, patients who completed palliative radiotherapy within 7 days of the first dose of trial treatment were excluded. Furthermore, delays in systemic treatment because of the use of radiotherapy should be avoided. Therefore, patients began receiving systemic therapy within 1 week of starting radiation therapy on our protocol.

The median PFS in the present study was similar to KEYNOTE-189 and 407,^22^^,^^23^ although several of our cases experienced long-term survival. The 12-month and 24-month PFS rates were 44.4% and 30.0%, respectively, and the 24-month and 36-month OS rates were 57.7% and 39.8%, respectively. The possibility that the relatively low proportion of PD-L1 TPS of less than 1% (16%) may have influenced the efficacy needs to be taken into account.

Cytotoxic agents are expected to have a synergistic effect with immunotherapy by inhibiting immunosuppressive cells, activating effector cells, or increasing immunogenicity and increasing T-cell infiltration.^24^ It has been suggested that cytotoxic agents may contribute to the short-term effect of avoiding early progression but may not have a synergistic effect on long-term survival.^25^, ^26^, ^27^ It was interesting to note that favorable 24-month and 36-month rates of OS were observed in the present study, but six patients (16.2%) experienced progressive disease within three months. Because the lesions prioritized for irradiation on protocol were those that required irradiation on the basis of clinical presentation, the most common site of radiotherapy was bone (52.6%). Therefore, it was possible that patients in poor condition because of painful bone metastases were recruited.

Thus far, stereotactic body radiation therapy (24 Gy in three fractions and 50 Gy in four fractions) has often been used in studies evaluating the abscopal effect in metastatic NSCLC.^10^, ^11^, ^12^^,^^15^, ^16^, ^17^ In murine breast and colon carcinoma models, in combination with CTLA-4 blockade, fractionated local radiotherapy of 24 Gy in three fractions was more effective than 30 Gy in five fractions for induction of the abscopal effect, associated with higher IFN-γ expression by T cells.^28^ In the pooled analysis described above, the abscopal response rate at 45 Gy in 15 fractions was inferior to both 24 Gy in three fractions and 50 Gy in four fractions and was similar to that of pembrolizumab alone. A reduction in the number of lymphocytes in blood was observed in the group receiving 45 Gy in 15 fractions.^21^ A retrospective analysis of three phase 1/2 trials found that the postradiotherapy absolute lymphocyte count may affect the occurrence of abscopal responses and thus influence prognosis in patients treated with radiotherapy and immunotherapy.^29^ Because 30 Gy in 10 fractions is usually indicated for palliative treatment, such as irradiating bone metastases in metastatic NSCLC, we considered that the 30 Gy irradiation dose was more feasible when combined with immunotherapy plus chemotherapy. The addition of 30 Gy in 10 fractions to pembrolizumab plus chemo reported promising efficacy in this study, but the appropriate radiation dose and fractions should be further investigated.

Regarding the safety profile, there was one grade 3 or higher radiation-mediated AE as judged by the attending physicians (esophagitis). The incidence of AEs (immune-related adverse event (irAE) and non-irAE) of grade 3 and higher was similar to that reported from the KEYNOTE-189 trial.^3^ Nevertheless, in this study, the incidences of all grades and grade 3 and higher irAEs were 44.7% and 26.3%, respectively. In KEYNOTE-189, these figures were 22.7% and 8.9%, respectively. Therefore, the incidence of irAEs in this study was higher than in the KEYNOTE-189 trial. The incidence of pneumonitis was 26.3% for any grade and 7.9% for grade 3 and higher. Among the 12 patients who received radiation to the lung or intrathoracic lymph nodes, five developed grade 1 or 2 pneumonitis, but no one developed grade 3 or higher pneumonitis. Of the nine patients irradiated mainly to the ribs or thoracic vertebrae, none developed radiation pneumonitis. The incidence of elevated liver enzymes was 34.2% for any grade and 13.2% for grade 3 and higher. Of the latter, two grade 3 and grade 4 increases were judged to be immune-mediated. None of these patients had received liver irradiation, so it was possible that the abscopal effect of the irradiation, rather than its direct effects, was responsible. Because the incidence of grade 3 or higher AEs was similar to that reported in the KEYNOTE-189 study, and there was only one treatment-related death, the safety profile was deemed tolerable.

The PEMBRO-RT trial reported that pneumonitis occurred in 26% of patients, with 11% having grade 3 and higher pneumonitis.^10^ Of these, 11% were considered immune-related, and no grade 3 and higher immune-related pneumonitis was observed. Another study conducted at the MD Anderson Cancer Center reported that pneumonitis occurred in 14% of patients in a cohort receiving traditional radiotherapy together with pembrolizumab (n = 21), with 10% being grade 3 and higher.^11^ Nevertheless, those trials included high proportions of patients with thoracic irradiation, and therefore, the results cannot be compared with ours. Mattes et al.^20^ conducted a single-arm prospective trial to assess whether radiotherapy can be added safely to ICI with or without chemotherapy. They found that patients undergoing combined ICI plus chemotherapy received a lower median biologically effective dose of stereotactic body irradiation than those undergoing ICI monotherapy but had a higher rate of radiation-induced toxicity.^20^ In the KEYNOTE-799 study, which was for locally advanced NSCLC, immune-mediated AEs occurred in 51 patients (45.5%) in cohort A (carboplatin, paclitaxel, and pembrolizumab) and 41 (40.2%) in cohort B (cisplatin, pemetrexed, and pembrolizumab). Grade 3 to 5 immune-mediated pneumonitis occurred in seven patients (6.3%) in cohort A and seven (5.9%) in cohort B. Four patients (3.6%) in cohort A and one (1.0%) in cohort B died owing to pneumonitis. These fatal AEs were attributed by the investigator to treatment with pembrolizumab, with the exception of one case of pneumonitis that was attributed to both pembrolizumab and radiotherapy.^13^ Our data and these previous reports suggest that the addition of radiation may increase the incidence of pneumonitis; thus, caution should be exercised. Lung V20 was limited to 10% or less in the PEMBRO-RT trial, whereas it was limited to 20% or less in our study. Safer thresholds for combination with immunotherapy need to be considered.

This study has some limitations. First, this was a single-arm study with a limited sample size. Therefore, randomized clinical trials comparing immunotherapy plus platinum-doublet chemotherapy versus adding radiotherapy to immunotherapy plus platinum-doublet chemotherapy are needed to answer the question of the necessity for radiation.

Second, our cohort has a single ethnicity because this study was conducted in Japan. Comparing global data and Japanese subset data of trials of pembrolizumab (KEYNOTE-189, 407, and 024), the incidence of irAEs, including pneumonitis, was higher in Japanese.^30^, ^31^, ^32^ Therefore, caution should be exercised when considering global applicability regarding the incidence of irAEs.

In conclusion, the addition of radiotherapy to pembrolizumab plus chemotherapy reported promising efficacy for patients with previously untreated metastatic NSCLC. Although caution should be exercised with regard to pneumonitis, AEs were deemed tolerable.

## CRediT Authorship Contribution Statement

**Yoko Tsukita:** Conceptualization, Data curation, Formal analysis, Investigation, Project administration, Visualization, Writing - original draft.

**Rei Umezawa:** Investigation, Writing - original draft.

**Taku Nakagawa:** Investigation, Writing - review & editing.

**Akira Anbai:** Investigation, Writing - review & editing.

**Tomonori Makiguchi:** Investigation, Writing - review & editing.

**Hisashi Tanaka:** Investigation, Writing - review & editing.

**Yosuke Horii:** Investigation, Writing - review & editing.

**Aya Suzuki:** Investigation, Writing - review & editing.

**Ryo Morita:** Investigation, Writing - review & editing.

**Hitomi Nogawa:** Investigation, Writing - review & editing.

**Hiroshi Yokouchi:** Investigation, Writing - review & editing.

**Nozomu Kimura:** Investigation, Writing - review & editing.

**Keiichi Jingu:** Conceptualization, Writing - review & editing.

**Akira Inoue:** Conceptualization, Writing - review & editing.

**Hisatoshi Sugiura:** Supervision, Writing - review & editing.

**Eisaku Miyauchi:** Conceptualization, Investigation, Project administration, Writing - review & editing.

## Disclosure

Dr. Tsukita reports grants to institution from Chugai Pharma and Eli Lilly; honoraria from AstraZeneca, Taiho Pharmaceutical, Eli Lilly, Merck Sharp & Dohme, Eisai, Chugai Pharmaceutical, Daiichi Sankyo, Bristol-Myers Squibb, and Nippon Boehringer Ingelheim; participation on an advisory board of AstraZeneca outside the submitted work. Dr. Nakagawa reports honoraria from Pfizer, Ono Pharmaceutical, Taiho Pharmaceutical, and Chugai Pharma outside the submitted work. Dr. Makiguchi reports honoraria from Asahi Kasei Pharma Corporation, Nippon Boehringer Ingelheim Co., Ltd., AstraZeneca, Eli Lilly, GlaxoSmithKline, Ono, Teijin Pharma, Taiho Pharmaceutical, and Chugai Pharmaceutical outside the submitted work. Dr. Tanaka reports honoraria from AstraZeneca, Chugai Pharmaceutical, Boehringer Ingelheim Japan, Pfizer Japan, Ono Pharmaceutical, and Bristol-Myers Squibb outside the submitted work. Dr. Morita reports honoraria from AstraZeneca, Bristol-Myers Squibb, Takeda, Chugai, Novartis, Pfizer, Daiichi Sankyo, and Amgen; participation on an advisory board of AstraZenea and Daiichi Sankyo outside the submitted work. Dr. Yokouchi reports grants to institution from Sanofi, AstraZeneca, Bristol-Myers, AbbVie, Takeda, Chugai, Daiichi Sankyo, Merck Sharp & Dohme, and Taiho; honoraria from AstraZeneca and Chugai Pharmaceutical outside the submitted work. Dr. Jingu reports grants to institution from Elekta, KK outside the submitted work. Dr. Inoue reports honoraria from AstraZeneca and Daiichi Sankyo outside the submitted work. Dr. Miyauchi reports grants from 10.13039/100010795Chugai Pharmaceutical Co Ltd., Eli Lily Japan KK, honoraria from Taiho Pharmaceutical Co Ltd., Bristol-Myers Squibb Co Ltd., Merck Sharp & Dohme KK, Ono Pharmaceutical Co Ltd., Daiichi Sankyo KK, Boehringer Ingelheim Japan Inc., Novartis Pharma KK, Kyowa Kirin Co Ltd., Merck Biopharma Co Ltd., Pfizer Inc., Eisai Co Ltd., Otsuka pharmaceutical Co Ltd., Amgen Inc., Thermo Fisher Scientific KK, Takeda Pharmaceutical Co Ltd., Nippon Kayaku Co Ltd., Sysmex Co, AstraZeneca KK, Chugai Pharmaceutical Co Ltd., and Eli Lily Japan KK outside the submitted work. The remaining authors declare no conflict of interest.
