# Supplementary material for: Phase 2 Trial of Combination Radiotherapy and Pembrolizumab Plus Chemotherapy in Patients With Previously Untreated Metastatic NSCLC: NJLCG 1902
Source: JTO Clin Res Rep. 2025 Feb 28;6(5):100817. doi: 10.1016/j.jtocrr.2025.100817 (PMC11992379; doi:10.1016/j.jtocrr.2025.100817)
Supplement: Supplementary Tables 1-3 [file mmc1.docx]

Supplemental Table 1. Response to treatment

|  | Total (n=37) | |
| --- | --- | --- |
|  | Number (%) | 95% CI |
| Partial response | 25 (67.6) | 50.2-82.0 |
| Stable disease | 8 (21.6) | 9.8-38.2 |
| Progressive disease | 3 (8.1) | 1.7-21.9 |
| Not evaluable | 1 (2.7) | 0.07-14.2 |
| Overall response rate | 25 (67.6) | 50.2-82.0 |
| Disease control rate | 33 (89.2) | 74.6-97.0 |

CI, confidence interval

Supplemental Table 2. Treatment exposures

| Regimen | Drug | Number of doses,  Median (range) |
| --- | --- | --- |
| Pembrolizumab/CDDP or CBDCA/PEM | CBDCA　or CDDP | 4 (1-4) |
|  | PEM | 6.5 (1-39) |
|  | Pembrolizumab | 7.5 (1-39) |
| Pembrolizumab/CBDCA/PTX or nab-PTX | CBDCA | 4 (2-4) |
|  | Nab-PTX or PTX | 10 (2-12) |
|  | Pembrolizumab | 4 (2-12) |

Abbreviations: CDDP, cisplatin; CBDCA, carboplatin; PEM, pemetrexed; PTX, paclitaxel

Supplemental Table 3. Progression-free survival subgroup analyses

| Variable |  | N | Median PFS (m)  (95%CI) | Hazard ratio  (95%CI) | *P* value |
| --- | --- | --- | --- | --- | --- |
| Age | ≥75 years | 7 | 8.12  (4.57-49.15) | 0.91  (0.34-2.44) | 0.8563 |
|  | <75 years | 30 | 8.38  (5.72-22.16) | Reference |  |
| Sex | Male | 28 | 8.38  (5.65-22.16) | 1.09  (0.44-2.73) | 0.8520 |
|  | Female | 9 | 8.12  (1.55-49.15) | Reference |  |
| ECOG PS | 0 | 22 | 11.34  (5.65-22.16) | 0.96  (0.43-2.15) | 0.9302 |
|  | 1 | 15 | 7.76  (2.99-NR) | Reference |  |
| Histology | Adenocarcinoma | 28 | 7.82  (5.72-41.62) | 0.83  (0.34-2.01) | 0.6749 |
|  | Squamous, NOS | 9 | 11.34  (2.99-16.18) | Reference |  |
| Disease stage | IVB | 24 | 7.63  (4.73-49.15) | 0.97  (0.43-2.18) | 0.9449 |
|  | Other | 13 | 13.08  (4.18-22.16) | Reference |  |
| PD-L1 | ≥50% | 14 | 16.18  (5.72-49.15) | 0.52  (0.22-1.21) | 0.1290 |
|  | Other | 23 | 7.82  (4.57-13.41) | Reference |  |
| irAE | ≥Grade 3 | 9 | 8.12  (5.72-22.16) | 0.96  (0.38-2.42) | 0.9331 |
|  | <Grade 2 | 28 | 13.08  (2.99-NR) | Reference |  |
| Radiation site | Bone | 19 | 7.76  (4.57-13.08) | 1.54  (0.69-3.44) | 0.2885 |
|  | Other | 18 | 13.41  (4.73-49.15) | Reference |  |

PFS, progression-free survival; CI, confidence interval; ECOG, Eastern Cooperative Oncology Group; NSCLC NOS, non-small cell lung cancer not otherwise specified; PD-L1, programmed death-ligand 1; irAE, immune-related adverse event.

Supplemental Table 4. Overall survival subgroup analyses

| Variable |  | N | Median OS (m)  (95%CI) | Hazard ratio  (95%CI) | *P* value |
| --- | --- | --- | --- | --- | --- |
| Age | ≥75 years | 7 | 23.11  (4.57-49.15) | 1.53  (0.54-4.30) | 0.4196 |
|  | <75 years | 30 | 34.09  (21.21-NR) | Reference |  |
| Sex | Male | 28 | 27.45  (16.37-NR) | 1.67  (0.54-5.16) | 0.3700 |
|  | Female | 9 | 35.84  (7.59-NR) | Reference |  |
| ECOG PS | 0 | 22 | 34.09  (22.26-NR) | 0.84  (0.33-2.16) | 0.7228 |
|  | 1 | 15 | 30.05  (5.13-NR) | Reference |  |
| Histology | Adenocarcinoma | 28 | 30.05  (21.21-NR) | 0.70  (0.25-1.99) | 0.5088 |
|  | Squamous, NOS | 9 | 27.45  (5.13-35.84) | Reference |  |
| Disease stage | IVB | 24 | 30.05  (16.37-NR) | 1.05  (0.39-2.81) | 0.9229 |
|  | Other | 13 | 27.45  (22.26-NR) | Reference |  |
| PD-L1 | ≥50% | 14 | 41.62  (16.37-NR) | 0.68  (0.26-1.81) | 0.4397 |
|  | Other | 23 | 27.45  (21.21-NR) | Reference |  |
| irAE | ≥Grade 3 | 9 | 27.45  (4.57-NR) | 0.93  (0.31-2.83) | 0.9043 |
|  | <Grade 2 | 28 | 34.09  (22.26-NR) | Reference |  |
| Radiation site | Bone | 19 | 30.05  (5.42-NR) | 1.40  (0.56-3.51) | 0.4693 |
|  | Other | 18 | 35.84  (22.26-NR) | Reference |  |

OS, overall survival; CI, confidence interval; ECOG, Eastern Cooperative Oncology Group; NSCLC NOS, non-small cell lung cancer not otherwise specified; PD-L1, programmed death-ligand 1; irAE, immune-related adverse event.

Supplemental Table 5. Patients with pneumonitis

| Pneumonitis  Grade | Radiation site | Lung V20* (%) |
| --- | --- | --- |
| 1 | Lung (primary) | 8.1 |
| 1 | Lymph node (right hilar) | 9.5 |
| 1 | Lung (primary) | 11.2 |
| 1 | Bone (acetabular) | - |
| 2 | Lymph node (mediastinal) | 1.6 |
| 2 | Adrenal grand | - |
| 2 | Lung (primary) | 6.5 |
| 3 | Bone (lumbar vertebrae) | - |
| 3 | Bone (acetabular) | - |
| 5 | Bone (cervical/lumber vertebrae, ilium) | - |

*Lung V20 data were collected for patients irradiated to the lung or intrathoracic lymph nodes.
